# Evaluating the economic impact of clinical pharmacist interventions in the women’s health setting in Qatar

**DOI:** 10.1007/s11096-025-01933-z

**Published:** 2025-06-07

**Authors:** Daoud Al-Badriyeh, Shimaa Aboelbaha, Nurhan ElShafey, Moza Al Hail, Wessam El-Kassem, Palli Valapila Abdulrouf, Binny Thomas, Islam Eljilany, Noor Alsalemi, Mariyam Syed, Dina Abushanab

**Affiliations:** 1 College of Pharmacy, QU Health, Qatar University, Doha, Qatar; 2 Department of Pharmacy, Drug Information Center, Hamad Bin Khalifa Medical City, Hamad Medical Corporation, Doha, Qatar; 3 Moffitt Cancer Center and Research Institute, Tampa, Florida USA; 4 Department of Obstetrics and Gynecology, Women’s Wellness and Research Center, Hamad Medical Corporation, Doha, Qatar; 5 Clinical Trial Unit, Hamad Medical Corporation, Doha, Qatar

**Keywords:** Adverse drug events, Clinical pharmacists, Cost, Economic impact, Interventions, Qatar, Women’s health

## Abstract

**Background:**

Adverse drug events (ADEs) resulting from nonoptimized medication therapy significantly drive-up healthcare costs. Clinical pharmacists are pivotal in managing medication regimens, effectively reducing these associated expenses.

**Aim:**

Given the scarcity of similar studies in the region, this study aimed to evaluate the overall economic impact of clinical pharmacists’ interventions against ADEs at the Women’s Wellness and Research Center (WWRC) in Qatar.

**Method:**

Analysis of the total economic benefit of clinical pharmacists’ interventions was performed through a retrospective chart review of patients’ records admitted during the periods of March 2018, July–August 2018, and January 2019. The current analysis was based on WWRC’s perspective, in which the cost savings and cost avoidance associated with the interventions were used to determine the total economic benefit. A sensitivity analysis was conducted to determine the robustness of the results. The study was approved at the main public healthcare provider.

**Results:**

A total of 331 interventions for 162 patients were included in the analysis. The total economic benefit was estimated to be QAR169,320 (USD46,503), comprising cost avoidance of QAR170,995 (USD46,964) and negative resource-use cost savings of QAR-1675 (USD-460). The increase in resource use was primarily due to the addition of other medications to therapy. The sensitivity analysis confirmed that the outcomes are robust, demonstrating a 100% probability for positive economic benefits in all simulated cases.

**Conclusion:**

Although there was an observed increase in resource utilization resulting from clinical pharmacists’ interventions, this study highlights their crucial role in mitigating the costs associated with preventable adverse drug events.

**Supplementary Information:**

The online version contains supplementary material available at 10.1007/s11096-025-01933-z.

## Impact statements

- The study supports integrating clinical pharmacists in women's health services, improving medication management and lowering drug-related problems.
- Results emphasise that pharmacist interventions prevent adverse drug events, indicating a need for policy adjustments to promote cost-effective pharmacotherapy and better resource allocation.
- The economic benefits of clinical pharmacy interventions encourage policymakers to utilize pharmacoeconomic data for strategies that improve medication safety and efficacy in women's health.

## Introduction

Drug-related problems (DRPs) significantly contribute to increased healthcare costs. A 2016 study predicted the annual cost of prescription drug-associated morbidity and mortality due to nonoptimized therapy at United States Dollar (USD) 528.4 billion [1]. Adverse drug events (ADEs), a common form of DRPs, are defined as unintended effects from medications resulting in harm or functional limitations [2]. The financial burden of ADEs is substantial, especially with the introduction of new medications and complex medical conditions [3, 4].

Focusing on women’s health is important due to unique medication management challenges. Women of childbearing age need a patient-centered approach that considers their medical histories and reproductive statuses to reduce DRPs, especially for pregnant or breastfeeding women who need careful evaluation of medication risks and benefits [5, 6]. Preventative measures, including clinical pharmacy interventions, are critical to reduce ADE risks [7].

An international review indicated that pharmacist services were cost-effective globally [8]. A study in China found early pharmacist consultations to be highly cost-effective, with an incremental cost-effectiveness ratio (ICER) of USD-475,499 per quality-adjusted life year gained [9]. Observational studies in France revealed that 84.3% of pharmacist interventions had significant clinical impacts, with 50% leading to cost reductions [10]. An economic analysis highlighted cost savings from pharmacists’ interventions for high-risk patients, with savings ratios between 181 and 584% [11].

Despite these benefits, there is limited evidence on the economic value of clinical pharmacy practices specifically for women of childbearing age. Globally, including in Qatar, healthcare systems aim to provide specialized care for unique populations. Integrating clinical pharmacists can optimize resources, enhance patient outcomes, and reduce healthcare costs [12–15].

Our study seeks to influence healthcare policies by demonstrating the vital role of pharmacists in women’s health services. To date, no studies have investigated the economic impact of pharmacist-led interventions in women’s health facilities. The Women’s Wellness and Research Center (WWRC), established in 2017, is Qatar's primary provider of secondary and tertiary women’s healthcare, offering specialized services within a modern framework [16]. Approximately 80% of healthcare services in Qatar are public, ensuring universal coverage, while some opt for private care [17].

### Aim

To evaluate the economic impact of clinical pharmacists’ interventions against ADEs for women in Qatar.

### Ethics approval

The study was approved by the Medical Research Center (MRC) of Hamad Medical Corporation (HMC), Qatar, MRC-01-19-110.

## Method

### Study settings

The study was conducted at the WWRC, at HMC [17]. In WWRC, clinical pharmacists are the healthcare providers concerned with performing pharmacotherapeutic follow ups with patients. This is usually achieved through reviewing patients’ charts and suggesting modifications to treatments as necessary. All clinical pharmacists are required to document clinical interventions as they are considered official proof of clinical pharmacists’ efforts to detect and resolve DRPs. A clinical intervention can be initiated by staff/operational (non-clinical) pharmacists; however, this it has to be through communication with the attending clinical pharmacists.

### Study design

The study was a retrospective review of the documented clinical pharmacists’ interventions in WWRC, which are defined as clinical pharmacist’s actions that directly resulted in alterations in patients’ care or therapy. All clinical interventions were directly sourced from the clinical intervention sheet, a component of each patient’s record in the Cerner electronic medical database. In cases where the clinical intervention sheet contained incomplete information, data was extracted from the patient’s file notes within the electronic medical records. The interventions were recorded by clinical pharmacists or clinical pharmacy specialists and subsequently validated by physicians. While staff pharmacists may propose interventions, these suggestions are documented through collaboration with the clinical pharmacists. The specific intervention categories defined in our system include: addition of another medication, discontinuation of a medication, switching to alternative medication, addition of a prophylactic agent during hospitalization, change in medication route, change in medication strength, change in medication dose, change in medication duration, change in medication frequency, addition of a lab test, addition of a serum level, and addition of a culture test.

### Study population

This study assessed clinical pharmacist interventions provided to inpatients admitted to WWRC. The inpatient population was female adults, 18 years or older, who were admitted to the WWRC inpatient wards.

The study sample was observed over three distinct months: March 2018, July 15 to August 15, 2018, and January 2019. The rationale behind selecting these specific months was to align with HMC annual performance evaluation period, which typically occurs in February. This timing could potentially impact the documentation practices of clinical pharmacists.

#### Inclusion criteria

- Women inpatients aged 18 years or older admitted to the WWRC during the specified data collection periods (March 2018, July 15 to August 15, 2018, and January 2019), including both obstetric and gynecologic patients.
- Patients had at least one medication indicated for continued use during hospitalization, including first-time treatments.

All interventions received physician approval to ensure alignment with clinical guidelines and patient safety.

#### Exclusion criteria

- Any interventions directly performed by a non-clinical pharmacist to maintain the integrity of clinical pharmacy practice.
- Interventions that were not approved by a physician, regardless of supporting evidence, to ensure that all recommendations align with clinical standards.

### Economic evaluation

#### Cost savings

Cost saving was defined as the reduced cost of therapy resource use because of clinical pharmacists’ interventions and was calculated based on a 3-month follow-up sampling duration. This was estimated by subtracting the total cost of therapy resource use “after” intervention from the total cost of therapy resource use “before” the interventions. Here, depending on what an intervention constitutes, the cost of a resource use can increase or decrease with the intervention. Further details on cost-saving calculations are demonstrated in Supplementary Table 1.

#### Cost avoidance

Cost avoidance was defined as the cost avoided because of preventing the occurrence of ADEs because of clinical pharmacy interventions, and it was calculated based on the 3-month study duration, by multiplying the cost of an ADE by the probability of the ADE happening. The cost of an ADE was estimated based on the assumption that it results in a 2-day additional hospital stay per patient, consistent with literature [12]. The estimation of the likelihood of ADE occurrence in the absence of intervention was calculated based on the Nesbit et al. method [18], and it used the following probabilities: 0 (none), 0.01 (very low), 0.1 (low), 0.4 (medium), or 0.6 (high). Further details on cost avoidance calculations and the Nesbit method are in Tables S1 and S2, respectively.

#### Total benefit analysis

Total benefit analysis was calculated as the sum of the total cost saving and total cost avoidance associated with all clinical interventions over a 3-month study follow-up period. The annual projected value of benefit was also calculated.

### Expert panel members

The probabilities of ADEs to occur in the absence of clinical interventions was determined by an expert panel utilizing the method suggested by Nesbit et al. [18] (in Supplementary Table 2). The panel was composed of four clinical pharmacists and one physician in WWRC. All had more than 5 years of clinical experience in the field. The probability of an avoided ADE was estimated for each intervention by each panel member, and an average was calculated.

### Perspective

The study was conducted from the perspective of the public WWRC hospital and, hence, only direct medical costs were used in the current analysis, excluding non-medical and indirect costs.

### Cost inputs

The monetary values of medication-based and non-medication-based resources were obtained from the pharmacy department as well as the finance and costing department. Using the Qatari Health Consumer Price Index (Trading Economics), all costs were adjusted for the financial year 2024 and were presented in Qatari Riyal (QAR) and USD.

### Sample size

Previous relevant literature research reports have not utilized a standardized sample size as this differs depending on variety of reasons including settings, context, and prevalence of the studied conditions. Economic evaluations, in contrast to clinical research, are concerned with making a cost estimate rather than testing hypotheses. Thus, even if underpowered, they still provide valuable information to guide decision-making [19]. It has been shown in relevant studies from the literature, especially those based on secondary/tertiary care hospitals [20], that the number of errors analyzed can range from less than 100 to less 500. On the basis of a preliminary investigation of the incidence of interventions in the study setting, we anticipated that a 3-month study period should provide sufficient time to collect more than 250 interventions.

### Statistical analysis

Descriptive statistics were used to analyze baseline demographics. Depending on their normal distribution, numerical data were presented as mean ± standard deviation (SD) or median ± interquartile range (IQR). Categorical variables were presented as frequencies and percentages. In order to test for any significant difference among patients’ characteristics over the 3-month follow up, Kruskal–Wallis and Chi-Square tests were used. Statistical significance was determined based on *p* values of < 0.05 and 95% confidence interval (CI). All Statistical tests were carried out using the IBM Statistical Package for Social Sciences (IBM SPSS®26 software).

### Sensitivity analysis

One-way sensitivity analysis was performed, with the targeted uncertain input of the cost of the ADEs, using an assigned ± 20% uncertainty range. Multivariate uncertainty analysis was performed by targeting the cost of the ADEs, using an assigned ± 20% uncertainty range, and probabilities of the avoided ADEs set by the expert panel, using an assigned ± 15% uncertainty range for any probability. Both sensitivity analyses were conducted via Monte Carlo simulation (1,000 iterations), using @Risk-5.7 (Palisade Corporation, NY).

## Results

### Characteristics of patients and interventions

A total of 331 interventions in 162 patients were documented during the study period. The median patient age was 35.5 ± 11 years. The general demographics were similar among the included patients of the three periods of follow-up, except for age; *p* = 0.002. The number of patients occurred during the July–August study period (n = 85, 52.4%). The majority of the included patients were Arabs (n = 45, 84.9%). All patients were admitted to inpatient care and did not require critical care. Details of the patient demographics can be seen in Supplementary Table 3. Subgroup analysis of demographics in the included population is also provided in Supplementary Table 4.

Overall, as per the categorization of interventions in the patient clinical intervention sheet, the most common interventions by the clinical pharmacists were related to the appropriateness of therapy (n = 169, 51.1%), followed by dosing and administration (n = 100, 30%), duplicate therapy (n = 41, 12.4%), contraindication and safety (n = 11, 3.3%), drug information (n = 8, 2.4%), and drug-drug interactions (n = 2, 0.6%). Details of the generated average probabilities of avoided ADEs that were associated with clinical pharmacy interventions are presented in Supplementary Table 5.

### Economic analysis

#### Cost savings

The added resource use cost associated with therapeutic interventions was estimated to be QAR25,986 (USD7,137), while the overall reduced resource use cost was QAR24,311 (USD6,677), adding to an overall cost saving that is in negative, i.e., QAR–1,675 (USD-460). The interventions that contributed the most to overall negative cost saving were the addition of other medications and the change in medications frequency. The interventions that contributed the highest to the increase in resource cost were the addition of another medication, and the change in medication route. The added and reduced costs with interventions as per different intervention type categories can be seen in Table 1.

**Table 1:** Added cost, reduced cost, cost avoidance, and total benefit analysis for clinical pharmacists’ intervention types

Type of intervention	Overall increased resource cost with interventions, QAR (USD)	Overall reduced resource cost with interventions, QAR (USD)	Overall cost avoidance, QAR (USD)
Addition of another medication	19,248 (5,287)	11,371 (3,123)	42,053 (11,550)
Discontinuation of a medication	0	8547 (2,341)	42,950.1 (11,796)
Switching to alternative medication	2964 (814)	649 (178)	16,781.1 (4,609)
Addition of a prophylactic agent during hospitalization	456 (125.2)	0	8070.3 (2,216)
Change in medication route	1918 (527)	851 (234)	7448.1 (2046)
Change in medication strength	0	0	1354.2 (372)
Change in medication dose	515 (142)	522 (143)	33,599 (9228)
Change in medication duration	1.6 (0.4)	2 (0.5)	1116.3 (307)
Change in medication frequency	52 (14)	2369 (651)	7411.5 (2035)
Addition of a lab test	452 (124)	0	5929.2 (1,629)
Addition of a serum level	80 (22)	0	3111(854)
Addition of a culture test	300 (82)	0	1171.2 (322)
Total	25,987 (7137)	24,311 (6677)	170,995 (46,964)

Outcomes of total benefit analysis	
Outcome	Value, QAR (USD)
Overall increased resource cost with therapy interventions per 3 months	25,986 (7137)
Overall reduced resource cost with therapy interventions per 3 months	24,311 (6677)
Overall cost savings per 3 months	− 1675 (− 460)
Overall cost avoidance per 3 months	170,995 (46,964)
Total benefit per 3 months	169,320 (46,503)
Projected total benefit per 1 year	677,280 (186,014)

QAR, Qatari Riyal; USD, United States Dollar

#### Cost avoidance

Presented in Supplementary Table 2, the probabilities of ADEs were calculated in the absence of clinical pharmacists’ interventions: no probability (0) was calculated for 2 interventions, very low probability (0.01–0.99) for 136 interventions, low probability (0.1–0.39) for 169 interventions, medium probability (0.4–0.59) for 25 interventions, and high probability (0.6) for one intervention. The overall cost avoidance due to the interventions over a 3-month period was QAR170,995 (USD46,964). The interventions that contributed the most to overall cost avoidance were change in medication dose, discontinuation of a medication and addition of another medication. Details of cost avoidance associated with each intervention category are presented in Table 1.

#### Total benefit analysis

Overall economic benefit in favor of the clinical pharmacist interventions is presented in Table 1, where the total economic benefit is calculated to be QAR169,320 (USD46,503) per patient and QAR143,579 (USD39,433) per intervention. The projected total benefit for one year was QAR77,280 (USD186,014).

### Sensitivity analysis

**Fig. 1:**
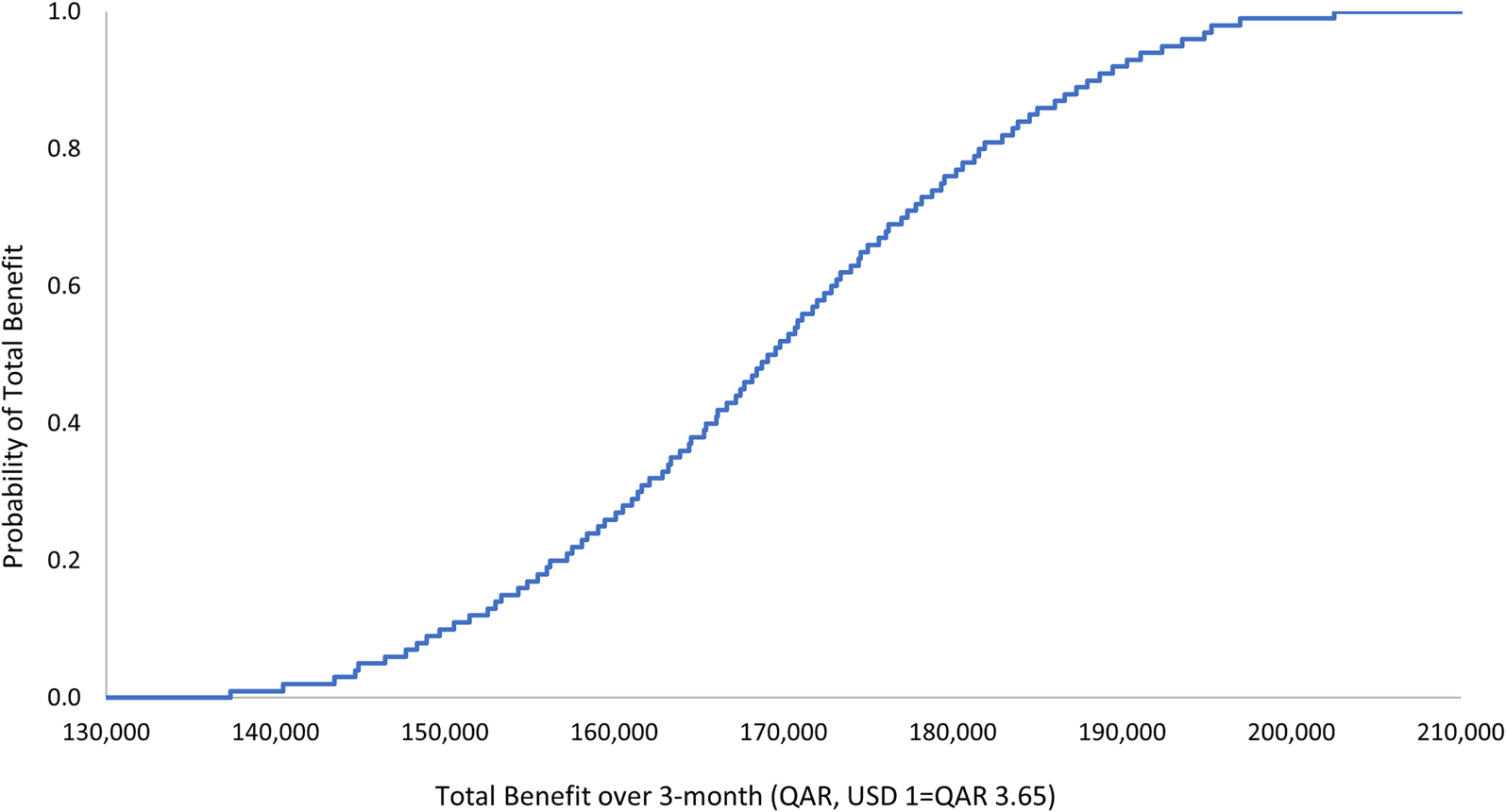
Total benefit probability curve over 3-month period (one-way sensitivity analysis)

**Fig. 2:**
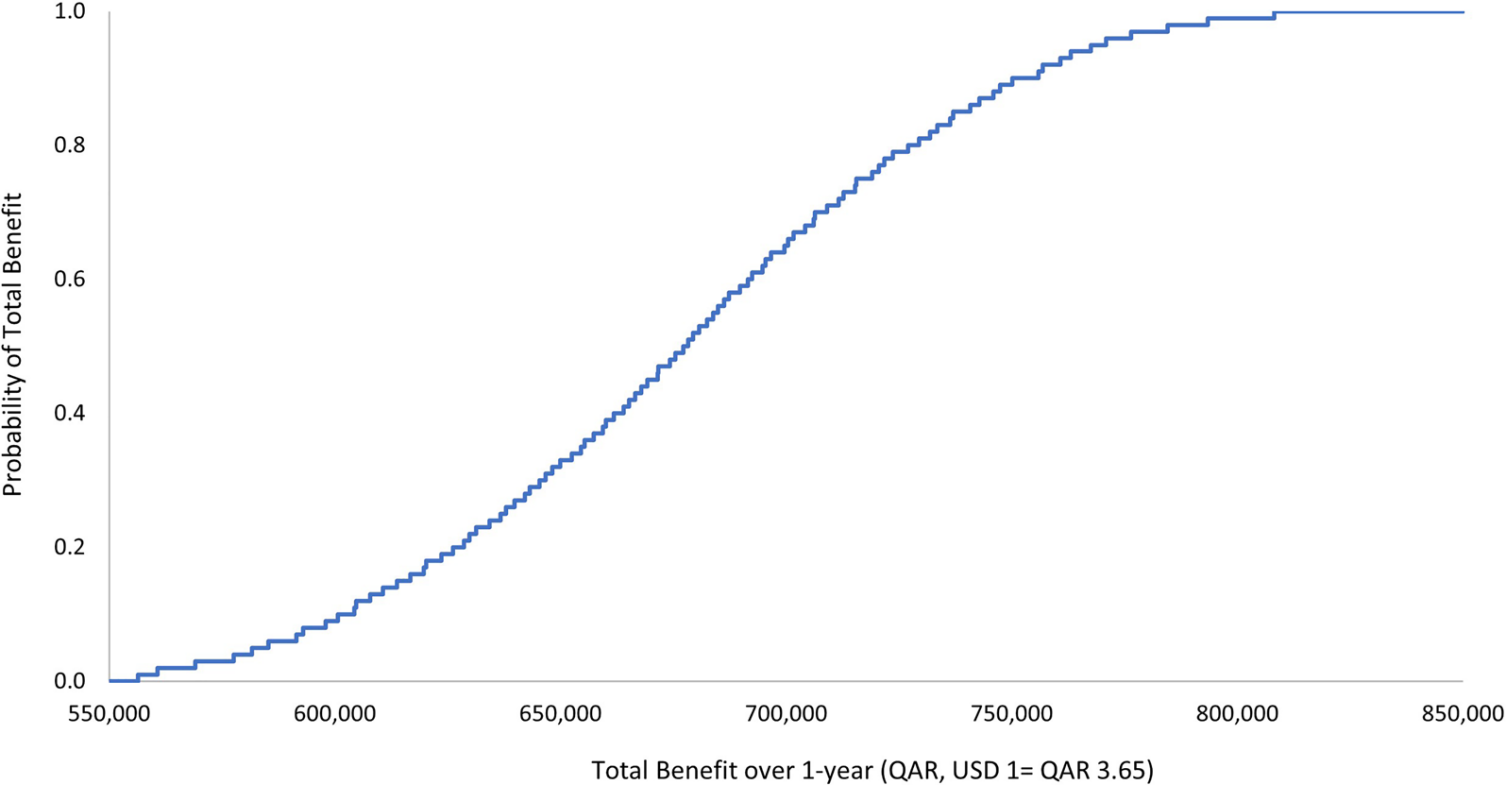
Total benefit probability curve over 1-year period (one-way sensitivity analysis)

**Fig. 3:**
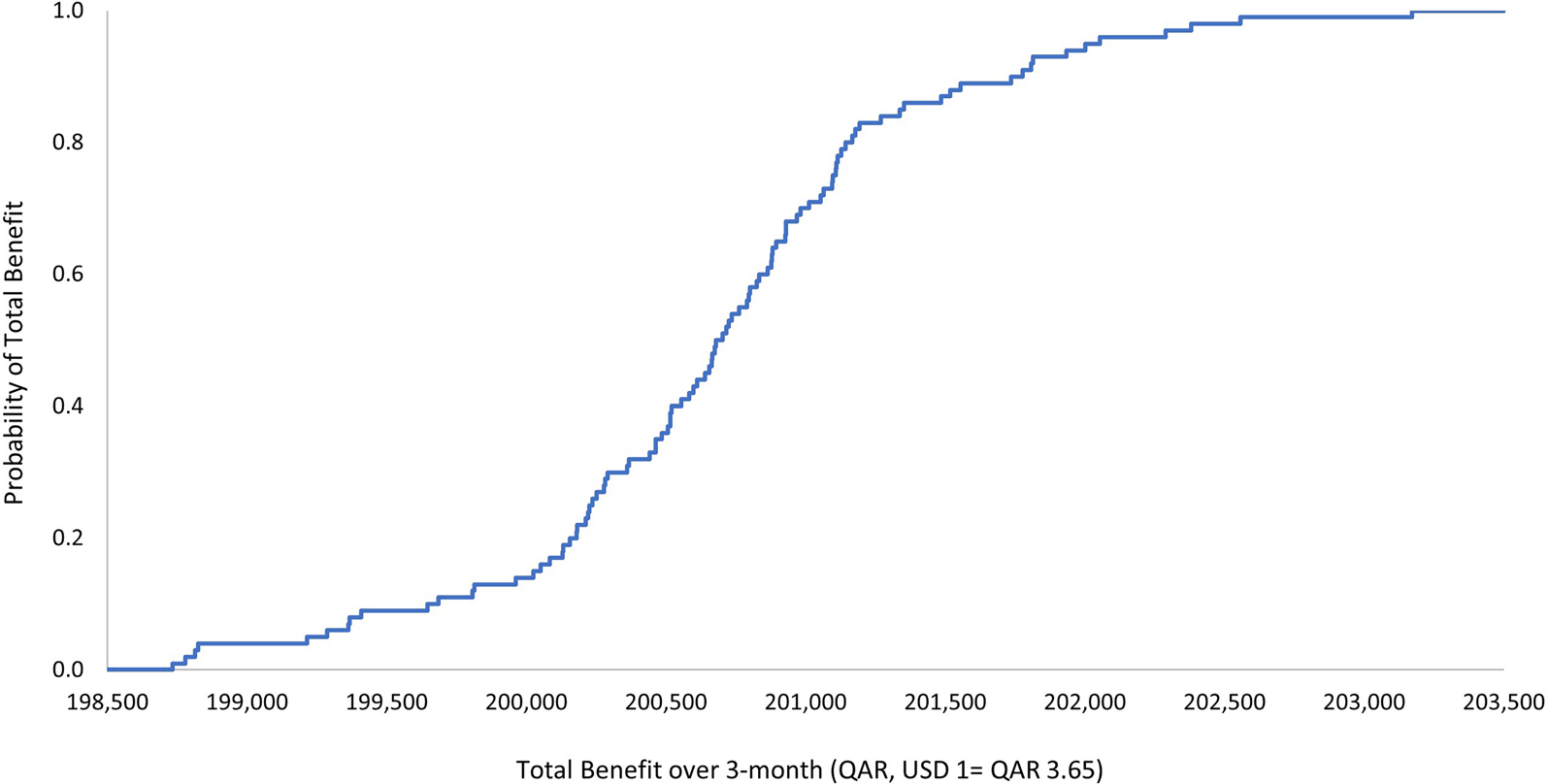
Total benefit probability curve over 3-month period (probabilistic sensitivity analysis)

**Fig. 4:**
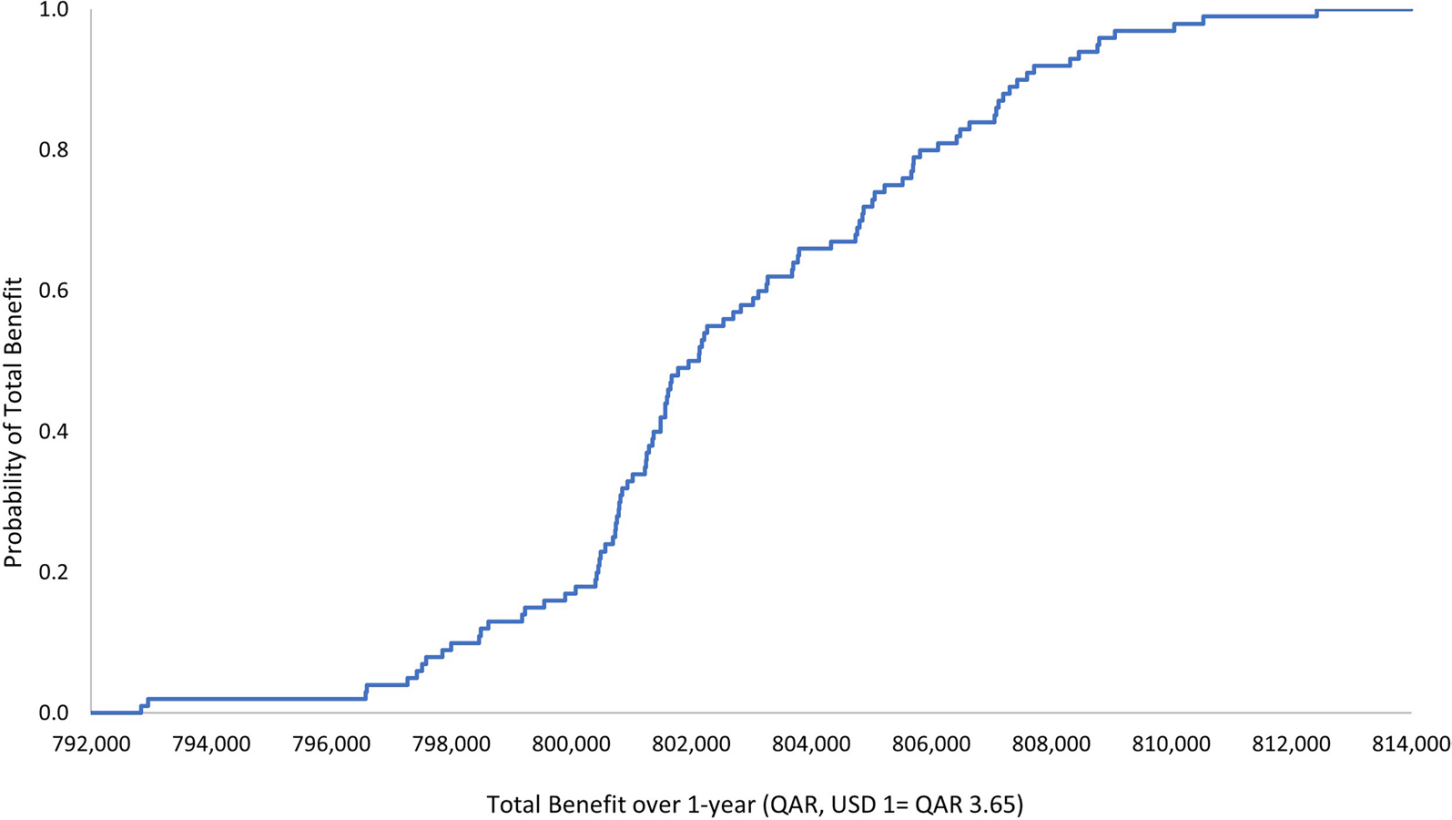
Total benefit probability curve over 1-year period (probabilistic sensitivity analysis)

**Fig. 5:**
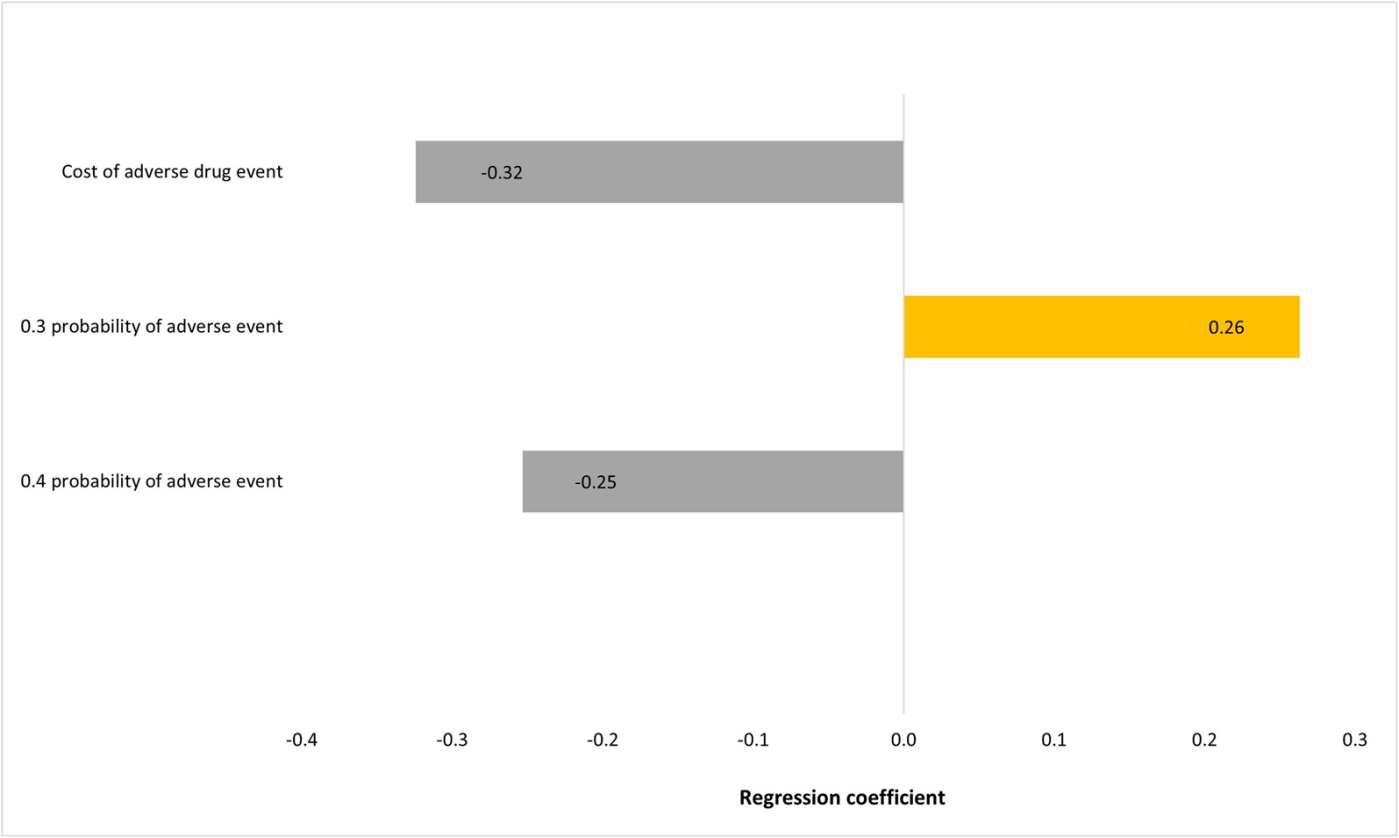
A regression tornado diagram of elements and their effect on the outcome

The outcome of the economic model was insensitive to uncertainty in the cost of the ADE. The one-way and multivariate sensitivity analyses demonstrated that there is a 100% probability that the pharmacist intervention is associated with a positive a total economic benefit. The ranges by which this economic benefit varies per the 3-monthly and annual durations are presented in Figs. 1, 2, 3, and 4. Input uncertainties and sampling distributions used in the one-way and multivariate sensitivity analyses, and the details of their outcomes, are shown in Supplementary Table 6. A regression Tornado analysis revealed that the main driver of the outcome was the cost of ADE, followed by the 0.3 probability of avoided ADE (Fig. 5).

## Discussion

### Statement of key findings

This study found that most clinical pharmacist interventions addressed DRPs related to therapy appropriateness. Interventions such as adding or switching medications primarily drove resource use costs, while cost avoidance was achieved through medication additions and discontinuations. Despite some negative cost impacts from these interventions, the overall projected annual benefit was QAR677,279 (USD186,015), driven by a significant reduction in preventable ADEs.

### Strengths and Weaknesses

This study is the first to assess the economic impact of clinical pharmacy interventions for DRPs in female inpatients at a major public women’s health facility in Qatar. However, limitations include a three-month data collection period, which may restrict the generalizability of findings and overlook long-term benefits like improved patient adherence. Literature supports that the advantages of clinical pharmacy interventions often extend beyond the short term, especially in chronic disease management [8]. The estimated probability of avoidable ADEs was based on assumptions. In line with other studies [12–15], the cost of ADEs was calculated based on the assumption that each ADE would result in an additional two days of hospitalization. Also, the expert panel’s probability estimates were closely tied to real-world data from WWRC, allowing for context-specific insights. Comprising two clinical pharmacists and a physician with over five years’ experience in women’s health, the panel ensured informed perspectives on patient care and complications. To reduce bias, pharmacists from both obstetrics and gynecology were included, and average estimates were validated by the physician for greater credibility. We acknowledge that variations in ADE severity can significantly affect cost estimates. However, sensitivity analyses were also conducted to address these uncertainties. While our approach was tailored and practical, we recognize that using a more structured method like the Delphi approach could improve future assessments by enhancing objectivity and consensus [21]. Lastly, excluding pharmacist-related costs may underestimate the economic impact.

### Interpretation

Our analysis of patient demographics showed notable variations in age, weight, and underlying conditions, all of which may have impacted the study’s findings. High rates of conditions such as labour pain and pregnancy-related conditions may have influenced the effectiveness of clinical pharmacy interventions. Older women typically have more complex medication regimens, potentially resulting in increased resource use but also greater opportunities for cost avoidance through effective management of DRPs.

Our results align with international literature demonstrating the cost-effectiveness of clinical pharmacy services. For example, studies conducted in several healthcare settings have shown that pharmacist interventions may generate significant cost savings by preventing medication-related complications and optimizing therapy [13–15, 22–24]. In contrast to our findings, which indicated an overall negative cost saving of QAR–1,675 (USD-460) due to added resource use costs associated with therapeutic interventions, previous studies have reported positive outcomes. For instance, a systematic review highlighted substantial cost reductions resulting from pharmacy services across different healthcare environments [8].

In our analysis, key contributors to increased costs were the addition of medications and changes in dosage or route. Although these costs appeared significant, they were justified by the substantial cost avoidance from preventing ADEs. Our findings indicate that the costs associated with preventing ADEs far outweigh those of the interventions, supporting the economic viability of pharmacist involvement. Sensitivity analyses affirmed a 100% probability that pharmacist interventions yield a positive economic benefit, confirming the robustness of our model.

Our study projected total economic benefit of QAR677,280 (USD186,014) per year for the entire patient population reflects a substantial positive outcome, even amidst the initial resource use costs. This contrasts with findings from other studies, such as those in Chile and Singapore, where clinical pharmacist interventions led to annual savings of USD263,500 and USD140,004, respectively, highlighting the variability in economic impact based on healthcare context [25, 26].

While some interventions initially increased costs, the long-term savings from preventing DRPs and ADEs validate the economic rationale for integrating clinical pharmacists into patient care teams.

In our analysis, the interventions that most frequently increased costs were the addition of another medication QAR19,248 (USD5,287), switching to alternative medication QAR2,964 (USD814), and changes in medication route QAR1,918 (USD527). While these costs may seem significant, they are justified by the substantial cost avoidance of QAR66,282 (USD18,205) from ADEs. Interventions such as the addition of another medication, switching to alternative medications, and changes in medication route were important in preventing complications that could lead to higher healthcare costs. The healthcare landscape increasingly emphasizes preventative care; although upfront costs may increase, the resultant savings from reduced hospitalizations yield a favorable return on investment. Both the one-way and multivariate sensitivity analyses demonstrated a 100% probability that pharmacist interventions yield a positive total economic benefit. This robustness shows that variations in ADE-related costs do not significantly impact the overall economic conclusions of our study. The main reason for this insensitivity lies in the substantial weight of avoided ADE costs in the total benefit calculation. Our findings revealed that the costs associated with preventing ADEs are significantly greater than the costs incurred from the interventions themselves. For example, the regression Tornado analysis identified that the cost of ADEs was the main driver of the economic outcome, followed by the probability of avoided ADEs. This suggests that even considerable fluctuations in the cost of ADEs do not alter the overarching conclusion that pharmacist interventions are economically beneficial. Furthermore, the high probability of avoided ADEs further reinforces the stability of our economic model. Since the interventions led to substantial cost avoidance due to the prevention of potential complications, the overall economic benefit remains robust against variations in individual cost parameters.

Qatar’s healthcare system aims to provide high-quality care while ensuring the sustainable and efficient use of resources, and our findings support this goal. By preventing ADEs and addressing other drug-related issues through targeted interventions, clinical pharmacists not only enhance patient safety but also reduce the financial burden associated with complications. The estimated cost avoidance of QAR170,995 (USD46,964) demonstrates a clear economic benefit that aligns with the government’s focus on optimizing healthcare expenditures. These interventions help contain costs related to prolonged hospital stays and additional treatments, reinforcing the efficiency of the healthcare system. As Qatar continues to advance its public health infrastructure, integrating clinical pharmacists will be pivotal in achieving national health strategy objectives, including improved health outcomes, increased patient satisfaction, and responsible resource utilization.

### Future research

Future studies with extended follow up periods are warranted to accurately capture the long-term economic effects of clinical pharmacists’ intervention on women’s health.

## Conclusion

Based on the study perspective and limitations, this study highlights the economically beneficial role of clinical pharmacists in managing inpatient care in women’s hospitals. Although their interventions increased resource use and costs, they significantly contributed to cost avoidance by preventing ADEs.

## Supplementary Information

Below is the link to the electronic supplementary material.

Supplementary file1 (DOCX 42 KB)
