# Supplementary material for: Evaluating the economic impact of clinical pharmacist interventions in the women’s health setting in Qatar
Source: Int J Clin Pharm. 2025 Jun 7;47(6):1720–9. doi: 10.1007/s11096-025-01933-z (PMC12630249; doi:10.1007/s11096-025-01933-z)
Supplement: Supplementary file 1 — Supplementary file1 (DOCX 42 KB) [file 11096_2025_1933_MOESM1_ESM.docx]

**Supplementary Table 1. Details of cost savings and cost avoidance calculations.**

| Cost of interventions (i.e. increased cost with interventions) | The increased cost of therapy with the intervention was (the cost of after-clinical pharmacy intervention therapy) minus (the cost of before-clinical pharmacist intervention therapy), when this is in +ve values. The cost of the before-clinical pharmacist intervention therapy was based on the assumed duration of the full course of the original therapy without the intervention, while the cost of the after-clinical pharmacist intervention therapy was based on the actual original therapy duration until intervention, added to the cost of the alternative therapy (therapy after the change) based on the duration of its full course. |
| --- | --- |
| Reduced cost with interventions | The reduced cost of therapy with the intervention was (the cost of before-clinical pharmacist intervention therapy) minus (the cost of after-clinical pharmacy intervention therapy), when this is in +ve values. The cost of the before-clinical pharmacist intervention therapy was based on the assumed duration of the full course of the original therapy without the intervention, while the cost of the after-clinical pharmacist intervention therapy was based on the actual original therapy duration until intervention, added to the cost of the alternative therapy (therapy after the change) based on the duration of its full course. |
| Cost avoidance | For each intervention with the potential to prevent an ADE, cost avoidance was calculated by multiplying the probability of an ADE in the absence of the intervention (estimated via the Nesbit et al. method) by the cost of an ADE. The overall cost avoidance was the sum of avoided costs with all interventions for DRPs. |

†HMC: Hamad Medical Corporation, ADE: adverse drug event, DRP: drug-related problem

**Supplementary Table 2. Adverse drug event probability description as per Nesbit et al. method**

| **Probability of ADE category** | **Probability score** | **Description** |
| --- | --- | --- |
| None | 0 | Information requested |
| Very low | 0.01 | For problem orders such as clarifications, missing information, or missing strengths) |
| Low | 0.1 | For preventing a potentially significant reaction, e.g., 2-4× normal dose, ineffective dose to produce therapeutic effect, or potential for therapeutic failure/toxicity due to incorrect schedule/route, duplicated therapy |
| Medium | 0.4 | For preventing a potentially serious reaction, e.g., allergy to an ordered drug, missing allergy information, 4–10× normal dose, no adjustment for renal or hepatic failure |
| High | 0.6 | For preventing a potentially fatal or severe reaction, e.g., 10× normal dose, narrow therapeutic index, life-threatening reaction) |

†ADE: adverse drug event

| **Supplementary Table 3. Demographic of the included population** | | | | | |
| --- | --- | --- | --- | --- | --- |
| **Total number of patients (n=162)** | | **March 2018**  **(n=24)** | **July-August 2018 (n=85)** | **January 2019**  **(n=53)** | **P value** |
| **Variable** | **Average  (±SD), or median (±IQR or, frequency (%)** |  |  |  |  |
| **Sex** | | | | | |
| Female | 162 (100%) | 24 (14.8%) | 85 (52.4%) | 53 (32.7 %) | N/A |
| Age | 35.5 ± 11 | 40.4 ± 8.7 | 36 ± 7.57 | 32 ± 10.5 | 0.002 |
| Weight | 79 ± 23 | 73.15± 21.75 | 82.74± 19.9 | 78.5 ± 19.65 | 0.143 |
| **Ethnicity** | | | | | |
| Arab | 137 (84.6%) | 21(87.5%) | 45 (84.9%) | 71 (83.5%) | 0.848 |
| Asian (non-Arab) | 20 (12.3%) | 2 (8.3%) | 7 (13.2%) | 11 (12.9%) |  |
| African (non-Arab) | 3 (1.9%) | 1 (4.2%) | 0 (0) | 2 (2.4%) |  |
| Others | 2 (1.2%) | 0 | 1 (1.9%) | 1 (1.2%) |  |
| **Underlying condition** | | | | | |
| Labour pain | 70 (43.2%) | 14 (20%) | 35 (50%) | 21 (30%) | 0.471 |
| Nausea and vomiting | 30 (18.5%) | 8 (26.7%) | 17 (56.7%) | 5 (16.6%) |  |
| Abdominal pain | 22 (13.6%) | 4 (18.2%) | 11 (50%) | 7 (31.8%) |  |
| C-section | 19 (11.7%) | 8 (42.1%) | 8 (42.1%) | 3 (15.8%) |  |
| Hypertension | 16 (9.9%) | 6 (37.5%) | 8 (50%) | 2 (12.5%) |  |
| Asthma | 3 (1.9%) | 2 (66.7%) | 1 (33.3%) | 0 |  |
| Ovarian cysts | 1 (0.6%) | 1 (100%) | 0 | 0 |  |
| H1N1 URTI | 1 (0.6%) | 0 (100%) | 1 | 0 |  |
| SD: standard deviation, IQR: interquartile range, URTI: upper respiratory tract infection | | | | | |

| **Supplementary Table 4. Subgroup analysis of demographics in the included population** | | | | | | | | | | | | | | | |
| --- | --- | --- | --- | --- | --- | --- | --- | --- | --- | --- | --- | --- | --- | --- | --- |
|  | | **Age and weight distribution**  **average (±SD), or median (±IQR)** | | **Ethnicity**  **(%)** | | | | **Underlying conditions**  **(%)** | | | | | | | |
| **Intervention type** | **Frequency (%)** | **Age** | **Weight** | **Arab** | **Asian (non-Arab)** | **African (non-Arab)** | **Others** | **Labour pain** | **N&V** | **AP** | **C-S** | **HTN** | **Asthma** | **OC** | **H1N1 URTI** |
| Addition of another medication | 90 (27.2) | 34.3 ± 10.1 | 76.3 ± 13.0 | 43 (26.5) | 5 (3.1) | 1 (0.6) | 0 | 24 (14.8) | 11 (6.8) | 7 (4.3) | 7 (4.3) | 3 (1.9) | 1 (0.6) | 1 (0.6) | 0 |
| Discontinuation of a medication | 87 (26.3) | 33.5 ± 9.4 | 75.7 ± 12.4 | 30 (18.5) | 7 (4.3) | 1 (0.6) | 2 (1.2) | 18 (11.1) | 6 (3.7) | 4 (2.5) | 1 (0.6) | 4 (2.5) | 0 | 0 | 0 |
| Switching to alternative medication | 33 (10) | 33.2 ± 9.0 | 83.0 ± 22.1 | 11 (6.7) | 5 (3.1) | 0 | 0 | 6 (3.7) | 4 (2.5) | 2 (1.2) | 3 (1.9) | 3 (1.9) | 1 (0.6) | 0 | 1 (0.6) |
| Addition of a prophylactic agent during hospitalization | 9 (2.7) | 36.5 ± 8.8 | 72.7 ± 18.1 | 3 (1.9) | 0 | 0 | 0 | 11 (6.8) | 5 (3.1) | 3 (1.9) | 5 (3.1) | 2 (1.2) | 1 (0.6) | 0 | 0 |
| Change in medication route | 22 (6.6) | 36.5 ± 10.2 | 78.0 ± 21.6 | 8 (5) | 1 (0.6) | 0 | 0 | 1 (0.6) | 0 | 1 (0.6) | 0 | 1 (0.6) | 0 | 0 | 0 |
| Change in medication strength | 4 (1.2) | 41.0 ± 7.3 | 88.8 ± 20.9 | 3 (1.9) | 1 (0.6) | 0 | 0 | 2 (1.2) | 0 | 1 (0.6) | 1 (0.6) | 0 | 0 | 0 | 0 |
| Change in medication dose | 50 (15.1) | 33.3 ± 10.6 | 76.0 ± 19.0 | 21 (13) | 0 | 1 (0.6) | 0 | 2 (1.2) | 2 (1.2) | 0 | 0 | 1 (0.6) | 0 | 0 | 0 |
| Change in medication duration | 2 (0.6) | 40.5 ± 9.5 | 75.2 ± 16.1 | 1 (0.6) | 0 | 0 | 0 | 3 (1.9) | 0 | 1 (0.6) | 0 | 0 | 0 | 0 | 0 |
| Change in medication frequency | 15 (4.5) | 36.5 ± 10.0 | 78.6 ± 24.8 | 5 (3.1) | 0 | 0 | 0 | 1 (0.6) | 2 (1.2) | 2 (1.2) | 0 | 0 | 0 | 0 | 0 |
| Addition of a lab test | 12 (3.6) | 32.2 ± 6.7 | 76.5 ± 13.4 | 6 (3.7) | 1 (0.6) | 0 | 0 | 2 (1.2) | 0 | 0 | 0 | 2 (1.2) | 0 | 0 | 0 |
| Addition of a serum level | 2 (0.6) | 38.0 ± 12.3 | 80.6 ± 21.0 | 2 (1.2) | 0 | 0 | 0 | 0 | 0 | 0 | 1 (0.6) | 0 | 0 | 0 | 0 |
| Addition of a culture test | 5 (1.5) | 33.6 ± 9.4 | 74.1 ± 17.8 | 4 (2.5) | 0 | 0 | 0 | 0 | 0 | 0 | 1 (0.6) | 0 | 0 | 0 | 0 |
| AP: abdominal pain, C-S: Caesarean- section, HTN: hypertension, N&V: nausea and vomiting, OC: ovarian cysts, URTI: upper respiratory tract infection | | | | | | | | | | | | | | | |

**Supplementary Table 5. Number of interventions, as per category, and the assigned probability of avoided adverse drug events**

| **Probability** | **Numbers and categories of the clinical interventions** |
| --- | --- |
| 0 | 2 interventions; “addition of another medication”, ‘switching to alternative medication’ |
| 0.005 | 9 interventions; ‘addition of another medication’, ‘Discontinuation of a medication’, ‘switching to alternative medication’, ‘increase in medication dose’ |
| 0.01 | 13 interventions; ‘increase in medication frequency’, ‘addition of another medication’, ‘change in medication strength’, ‘change in medication route’, ‘decrease in medication dose’, ‘Discontinuation of a medication’, ‘switching to alternative medication’ |
| 0.05 | 9 interventions; ‘addition of another medication’, ‘Discontinuation of a medication’, ‘increase in medication dose’, ‘Requesting a lab test’, ‘switching to alternative medication’, |
| 0.055 | 100 interventions; ‘increase in medication frequency’, ‘increase in medication dose’ ‘addition of another medication’, ‘change in medication strength’, ‘change in medication route’, ‘decrease in medication dose’, ‘Discontinuation of a medication’, ‘switching to alternative medication’, ‘Requesting a lab test’, ‘ addition of a culture test’ |
| 0.03 | 3 interventions; ‘requesting a lab test’, |
| 0.1 | 76 interventions; ‘discontinuation of a medication’, ‘addition of another medication’, ‘switching to alternative medication’; ‘increase in medication duration’, ‘change in medication route’, ‘increase in medication frequency’, ‘change in medication strength’, ‘increase in medication dose’, ‘decrease in medication duration’, ‘decrease in medication frequency’, ‘decrease in medication dose’, ‘requesting a lab test’. |
| 0.2 | 4 interventions; ‘increase in medication dose’, ‘decrease in medication dose’, ‘decrease in medication frequency’. |
| 0.205 | 20 interventions; ‘switching to alternative medication’, ‘addition of another medication’, ‘change in medication route’, ‘decrease in medication frequency’, ‘Discontinuation of a medication’, ‘requesting a lab test’. |
| 0.25 | 63 interventions; ‘addition of another medication’, ‘Discontinuation of a medication’, ‘addition of a prophylactic agent during hospitalization’, ‘decrease in medication dose’, ‘requesting a lab test’, ‘switching to alternative medication’, ‘increase in medication dose’, ‘decrease in medication frequency’, ‘change in medication route’, ‘increase in medication duration’, ‘decrease in medication dose’. |
| 0.305 | 1 intervention; ‘decrease in medication dose’ |
| 0.35 | 5 interventions; ‘addition of another medication’, ‘requesting a lab test’, ‘decrease in medication dose’, ‘Requesting a serum level’. |
| 0.4 | 23 interventions; ‘discontinuation of a medication’, ‘decrease in medication dose’, ‘addition of another medication’, ‘addition of a prophylactic agent during hospitalization’, ‘decrease in medication frequency’, ‘switching to alternative medication’. |
| 0.5 | 2 interventions; ‘increase in medication dose’, ‘Requesting a serum level’. |
| 0.6 | 1 intervention; ‘discontinuation of a medication’ |

| **Supplementary Table 6. Uncertainty distributions used for one-way and probabilistic sensitivity analyses, and their outcomes.** | | | | |
| --- | --- | --- | --- | --- |
| **Variable** | **Point estimate, QAR (USD)** | **Variation range, QAR (USD)** | **Projected total benefit per 1 year range, QAR (USD)** | **Total benefit per 3-month, QAR (USD)** |
| **One-way sensitivity analysis** | | | | |
| Cost of adverse drug event | 1,830 (502) | Triangular distribution,  QAR 1,464, 1,830, 2,196  (USD 402, 502, 603) | Mean:  677,315  (186,024), 95% CI  556,433  to 808,164  (152,824 to 221,962) | Mean:  169,347  (46,511), 95% CI  137,324 to 202,516  (37,716 to 55,621) |
| **Multivariate uncertainty analysis** | | | | |
| Very low probability for ADE | 0.03 | Triangular distribution  (0.025,0.03,0.035) | Mean:  802,723  (220468), 95% CI  792,826  to  812,422  (217,750 to 223,132) | Mean:  200,679  (55,117), 95% CI  198,734  to  203,170  (54,582 to 55,801) |
| Low probability for ADE | 0.1 | Triangular distribution  (0.09,0.1,0.11) |  |  |
| Low to moderate probability for ADE | 0.2 | Triangular distribution  (0.17,0.2,0.23) |  |  |
| Low to moderate probability for ADE | 0.3 | Triangular distribution  (0.26,0.3,0.35) |  |  |
| Moderate probability for ADE | 0.4 | Triangular distribution  (0.35,0.4,0.46) |  |  |
| Moderate to high probability for ADE | 0.5 | Triangular distribution  (0.43,0.5,0.58) |  |  |
| †QAR: Qatari Riyal, USD: United States Dollar, CI: confidence interval | | | | |
